# Dielectric Spectroscopy of Pressurized *Saccharomyces cerevisiae*

**DOI:** 10.1007/s11483-014-9367-y

**Published:** 2014-09-17

**Authors:** Szymon Starzonek, Małgorzata Rutkowska, Sylwester J. Rzoska, Aleksandra Drozd-Rzoska, Monika Fonberg-Broczek, Barbara Sokołowska, Julio C. Martinez-Garcia

**Affiliations:** 1 Institute of Physics & Silesian Intercollegiate Center for Education and Interdisciplinary Research, University of Silesia, ul. 75 Pułku Piechoty 1A, 41-500 Chorzów, Poland; 2 Institute of High Pressure Physics, Polish Academy of Sciences, ul. Sokołowska 27/39, 01-142 Warsaw, Poland; 3 Prof. Wacław Dąbrowski Institute of Agricultural and Food Biotechnology, Departmentof Fruit and Vegetable Product Technology, ul. Rakowiecka 36, 02-532 Warsaw, Poland; 4 University of Berne, Freiestrasse 3, Berne, CH-3012 Switzerland

**Keywords:** High pressures, Dielectric spectroscopy, Foods, Yeast

## Abstract

Results of broadband dielectric spectroscopy (BDS) in *Saccharomyces cerevisiae* (baker’s yeast), *in situ* as the function of pressure are presented. They show a clear evidence of a threshold to the new pattern of the pressure evolution of the static dielectric permittivity and DC electric conductivity already for *P*_*t*_ ≈ 200*MPa* at *T* = 5^*o*^ *C* and *P*_*t*_ ≈ 300*MPa* at *T* = 25^*o*^ *C*. BDS monitoring versus pressure tests up to *P* = 400*MPa* revealed particularly notable changes of properties after 30 minutes of compressing. Finally, the correlation between the amount of the spectrophotometric maximum absorbance and the DC electric conductivity was found. All these indicate significance of BDS as the tool for testing of pressure properties of cells assemblies, model foods etc., *in situ* under high pressures.

## Introduction

The broadband dielectric spectroscopy (BDS) is considered as a promising method for monitoring biological properties of cells [1–4]. The BDS monitoring can give the information on membrane capacitance, cytoplasmic conductivity and permittivity and the cell shape and structure. The effect of physical and chemical treatments on the cellular membrane can also be detected, including the degree of the disruption of cells walls [1–4]. Regarding practical implementations of BDS in the industry area, the possibility of monitoring of cell concentration in cultivation and fermentation is worth mentioning, along with the possibility of the use of these data for the estimation of the cell cycle [5].

These applications are notably strengthened by unique features of the modern BDS which can detect, in a single scanning process, up to 15 decades in frequency/time. Moreover, modern BDS spectrometers can operate with high resolution in a very broad range of electric capacitances and conductivities, which is particularly important for bio-samples [6]. Nowadays, the temperature and concentrational (isothermal) BDS behavior of diluted and dense solutions of cells is relatively well evidenced experimentally, although models for the analysis of experimental data are still puzzling [1–4]. This can be associated with extraordinary complexity of “living and active bio-colloids/bio-composites” [7].

One of notable gaps in experimental BDS studies on bio-systems is the practical lack of results related to the impact of hydrostatic pressure [6]. There is a general difference between the application of pressure and temperature. The latter influences the activation energy whereas the compression changes the density, the free volume and distances between species in a fluid system. On the practical side, significance of the pressure-related insight into properties of bio-systems indicates the boost of high pressure preservation (HPP) of foods technology, known also as the “cold pasteurization”, pascalization or bridgemanization [8–10]. For basic HPP industrial implementations the high pressure pulse (300–600 MPa) is applied for 3–10 minutes. This can lead to the reduction of the count of microorganisms from 5 to 8 decades [8–11]. The high microbiological safeness of HPP-treated foods is inherently associated with a set of highly beneficial features [6–10]: (i) the extension of shelf-life from 1 to 3 to even 90 days, (ii) taste, flavor and texture of the fresh product, (iii) preservation of vitamins amounts, (iv) avoiding of chemical preservatives, (v) the method can be applied for already packed food, (vi) the application is not limited to fluids, (vii) HPP is an environment-friendly method, i.e. it needs much less energy than the thermal pasteurization and there is practically no waste.

These unique advantages of the HPP technology coincides with expectations of the 21st century consumers with regard to high quality and healthy food products. Nowadays, there are over 200 large industry-scale HPP processors with the internal volume of the pressure chamber from 100 to 300 L. Their number increase by ca. 17 % each year which indicates the potential of HPP world foods market [11]. Notwithstanding, the fundamental biophysical base of HPP-related issues is still very limited. One of the most important cognitive gaps is related to studies *in situ* under high pressure. The comprehensive insight into properties of biosystems and foods in the pressure-temperature (*P*-*T*) plane can yield a base for a pressure-related modelling of novel foods. BDS offers unique research possibilities for *in situ* tests under high pressure due to existing state-of-the-art experimental implementations developed during studies on soft matter systems [12–17].

This report presents results of the first ever *in situ* high pressure BDS investigations for *Saccharomyces cerevisiae* (baker’s yeast), the unicellular eukaryotes which are widely used in biotechnological model processes [8–10].

## Experimental

**Fig. 1:**
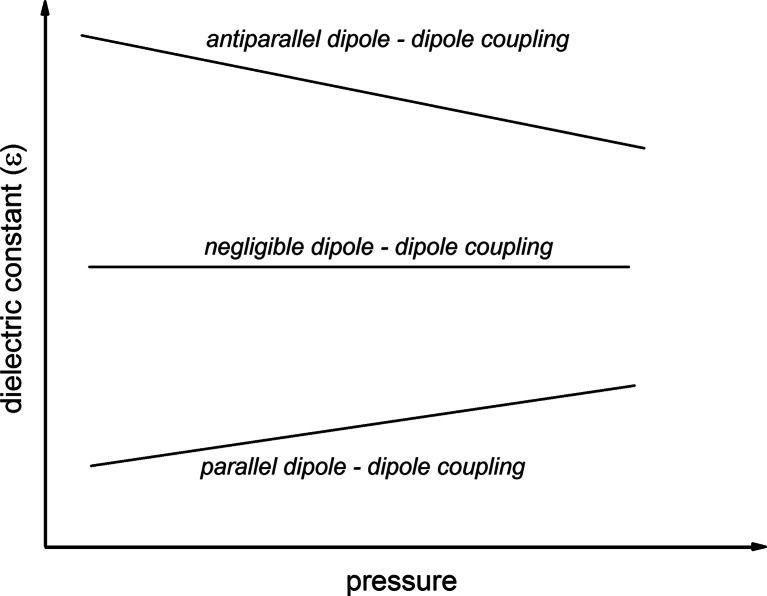
Pressure dependences of dielectric constant showing preferable type of dipole – dipole couplings in fluid dielectrics

1. ***Preparation of S. cerevisiae model suspensions.***
  Suspensions of *S. cerevisiae* NCFB 3191 strain were used. Twenty-four hour culture in YPG-broth (1 % yeast extract – Difco, 1 % peptone – Difco, 2 % glucose – POCh) was centrifuged at 4 ° C for 10 min at 6000 × g, and the sedimented cells were aseptically re-suspended into phosphate-buffered saline (PBS, pH 7.2) and again centrifugated. The washing procedure was repeated twice more. The final suspensions of *S. cerevisiae* were prepared in PBS, reaching cell concentration of about 10^19^–10^20^ cfu/ml (colony forming units/ml, ie. living cells/ml).
2. ***Electron microscopy.***
  Cells after UHP treatment were centrifuged 8 min at 3500 × g, washed carefully with distilled water and again centrifuged under the same conditions. The cells was suspended in 2 % solution of glutaraldehyde and stored over night at room temperature. The samples were centrifuged for 10 min at 3500 × g and the cells were prepared for electron microscopy. Ultrathin sections were observed under a transmission electron microscope JEM 100C (JEOL, Tokyo, Japan). The cells treated at 300 and 400 MPa showed essential ultrastructural changes. About 80 % of cells population, showed cell wall disruption and electron-dense areas of amorphous material packed with crushed microsomes and ribosomes.
3. ***UV analysis.***
  For the spectroscopic studies, UV-visible 1800 Shimadzu double beam spectrophotometer was used to record the spectra. The solvent used for the assay was spectroscopic-grade phosphate saline buffer. The yeast samples after high pressure treatment were centrifuged at for 20 min at 6000 × g, and the supernatants obtained were scanned in the 240–280 nm UV region. The wavelength maximum was observed at 260 nm, which relates to the yeast cell nucleotides and aromatic amino-acids present in the supernatant as the result of the high pressure cell wall disruption [8].
4. ***High pressure treatment.***
  The precise scheme of the high pressure set-up is given ref. [16]. It enabled changes of pressure up to *P* = 500 MPa, monitored via the tensometric pressure meter with precision + 0.1 MPa. The temperature of the pressure chamber was yielded via a large volume (25 Liter) thermostat with external circulation, enabling the temperature stabilization up to 0.02 K. Temperature was measured via constantant-copper thermocouples inside the pressure chamber. Additionally w thermocouples scanned the temperature gradient along the chamber. Pressure was measured with the precission ±0.1 MPa using tensometric pressure meter Nova Swiss. The sensor was located near but outside the pressure chamber, to avoid any impact of temperature on measurements.
  The greatest challenge for any *in situ* measurements under high hydrostatic pressure is the total isolation of the tested sample from the pressurized medium, matched with forceless transmission of the pressure to the sample. Moreover, multi-cycle increasing and decreasing of the applied pressure should be possible. Finally, the tested samples should be in contact only with chemically inert materials. The authors of the given paper developed the high pressure electric capacitor fulfilling all these conditions. Its scheme is given in ref. [16]. The flat-parallel measurement capacitor applied in the given research had the gap *d* = 1 mm and diameter 2*r* = 16 mm). Tested samples were in contact only with the stainless steel, quartz and Teflon. The pressure is transmitted from the pressurized liquid to the sample via the deformation of 50*μm* stretched Teflon film. The presented in ref. [16] unique construction of the capacitor is very solid and does not enable changes of any significant part during pressurization. It is notable that its body is made from Invar, hence it is also not sensitive to temperature changes.
5. ***Broadband dielectric spectroscopy under pressure.***
  For BDS studies the Alpha-A BDS Novocontrol (Montabaur, Germany) analyzer (model year 2013), yielding an insight for frequencies *f* = 0.001 Hz to 10 MHz (9 decades) with 5–6 digits resolution was used. Generally, BDS yields the complex dielectric permittivity *ε* ^∗^(*ω*) = *ε* ' (*ω*) + *iε* ^' '^(*ω*), *ω* = 2*πf*. The first component can be determined as *ε* ' (*f*) = *C*/*C*_0_, where *C*_*0*_ and *C* are electric capacitances of the measurements capacitor and the capacitor with the tested dielectric sample. The second component is experimentally defined as *ε* ' ' (*ω*) = 1/*ωRC*_0_ = *ε* '/*ωRC*, where *R* denotes the resistivity. For the so-called static domain, in liquids most often for 1*kHz* < *f* < 1*MHz*, *ε* ' (*f*) is constant and referred to as dielectric constant (*ε* ' (*f*) = *ε*). In this domain the temperature and pressure evolution of the dielectric constant allow to identify the dominating type of arrangement of permanent dipole moments [6–17]. The identification related from the pressure evolution is presented in Fig. 1. The temperature case is discussed in ref. [17].

When lowering the frequency values of *ε* ' (*f*) permanently increase, since the low – frequency electric field can interact with large and inert ions, in dielectric and molecular physics often recalled as “ionic impurities” [6, 17]. The translation of different types of “ionic impurities” present in a fluid dominates the BDS output in this region. In the “ionic impurities” dominated region it is convenient to transform experimental data to the complex electric conductivity form *σ* ^∗^(*ω*) = *iωε*_0_ *ε* ^∗^(*ω*) = *σ* ' (*ω*) + *iσ* ' ' (*ω*) = *ωε*_0_ *ε* ' ' (*ω*) + *ωε*_0_(*ε* ' (*ω*) − 1), where the electric constant (vacuum permittivity, permittivity of free space) *ε*_*o*_ ≈ 8.854*pF*/*m* The tradition and practical reasons caused that in dielectrics physics and in soft matter physics for (*ε* ', *ε* ' ') relative and dimensionless values are used. As visible from the above definition this is not the case of (*σ* ', *σ* ' '). Usually, in liquids and soft matter systems in the region dominated by electric charges motion *ε* ' (*ω*) is not affected but *ε* ' (*ω*) increases with decreasing frequency *ω* = 2*πf* in such a way that the slope at the plot log_10_ *ε* ' ' (*ω*) vs. log_10_ *ω* is equal to − 1.. Consequently, in this domain *σ* ' (*ω*) is independent from changes of the frequency and called DC conductivity (*σ*_*DC*_) and often simply electric conductivity. The DC “static” electric conductivity can be considered as the metric of translation of “ionic dopants” and the static dielectric permittivity *ε* = *ε* ' (*f*) ≈ *const* gives an insight into polarization and permanent dipoles arrangements [17]. The temperature or pressure dependence of *σ*_*DC*_ gives information on the conductivity mechanism.

The imaginary part of dielectric permittivity *ε* ' ' (*f*) is associated with energy losses and offers and insight into relaxation processes, manifesting as peaks in *ε* ' ' (*f*) spectrum. In the low frequency and “ion-dominated” region one should expect its growing up described by *ε* ' ' (*f*) ∝ *f* ^− *ϕ*^ with *ϕ* = 1 dependence, in agreement with the definition of DC electric conductivity above [6].

In “classical” molecular liquids one should expect that the DC electric conductivity will decrease with increasing pressure. In the simplest case, for describing such process the Barus equation [18], can be used: *σ*(*P*) = *σ*_0_^*P*^ exp(−*V*_*a*_ *P*), where *V*_*a*_ denotes the constant activation volume and *σ*_0_^*P*^ is the prefactor related the value of *σ*(*P*) for *P* = 0. It can be considered as the pressure counterpart of the simple Arrhenius relation *σ*(*T*) = *σ*_0_ exp(−*E*_*a*_/*RT*), where *R* denotes the gas constant and *E*_*a*_ is the activation energy. In a limited range of pressures, the Barus equation can be reduced to the linear dependence *σ*(*P*) = *σ*_0_ exp(−*V*_*a*_ *P*) ≈ *σ*_0_(1 − *V*_*a*_ *P* + …) = *σ*_0_ − (*σ*_0_ *V*_*a*_)*P*, due to the implementation of the Taylor expansion. Such approximation is valid only for relatively small values of the argument (pressure P in this case), If for experimental data notable distortion from such simple relation appears one should use the functional, experimental form (preferably).

This report bases on *in situ* measurements of *ε* ^∗^(*f*) for selected pressures and the discussion of the pressure evolution of dielectric constant *ε*(*P*), DC conductivity *σ*(*P*) and *ε* ' ' (*f*, *P*) spectra. BDS studies has been carried out for high concentration of *Saccharomyces cerevisiae* (10^19^–10^20^ cells/ml) to avoid a significant influence of the Maxwell–Wegner (MW) effect [6] which can lead to the parasitic polarization of the capacitor plates and which can notable bias output results. The key target of given studies was the preliminary test of the possibility of BDS technique as the tool for monitoring the high pressure *cold pasteurization*-related processes *in situ*.

## Results and Discussion

**Fig. 2:**
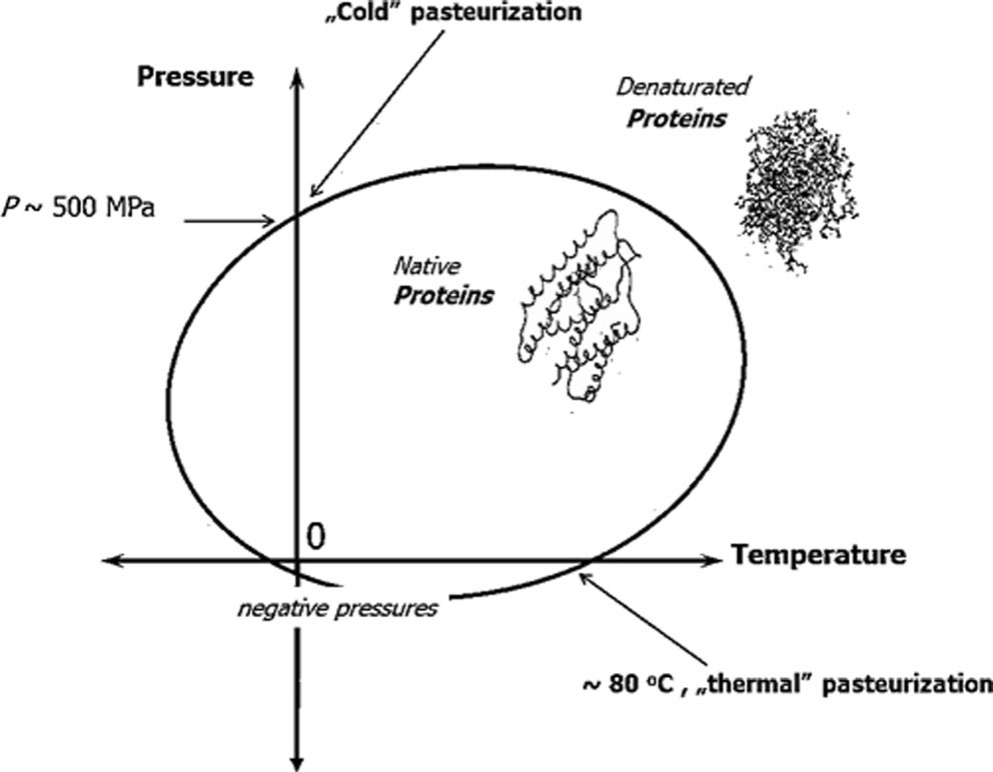
The schematic presentation of proteins denaturation in the pressure – temperature (P-T) plane [8, 19]. The form of a protein prior and after the denaturation process is shown

Generally, high pressures acts on living bio-systems in two ways. First, both heating and compressing can lead to the denaturation of proteins. This is related the to the classic or “cold” pasteurization. Second, on the higher level of complexity, one can expect the interruption of cell walls. The high pressure denaturation in the *P*-*T* plane is portrayed by an elliptic-like curve, crossing the *P* ≈ 0.1*MPa* (atmospheric pressure) line near *T* ≈ 80^*o*^ *C*, related to pasteurization [8, 19]. The isothermal “cold” (i.e. well below the thermal pasteurization limit) compression, for 5^*o*^ *C* < *T* < 40^*o*^ *C*, leads to the denaturation at 400 < *P* < 600*MPa* [6]. This behavior is schematically shown in Fig. 2.

**Fig. 3:**
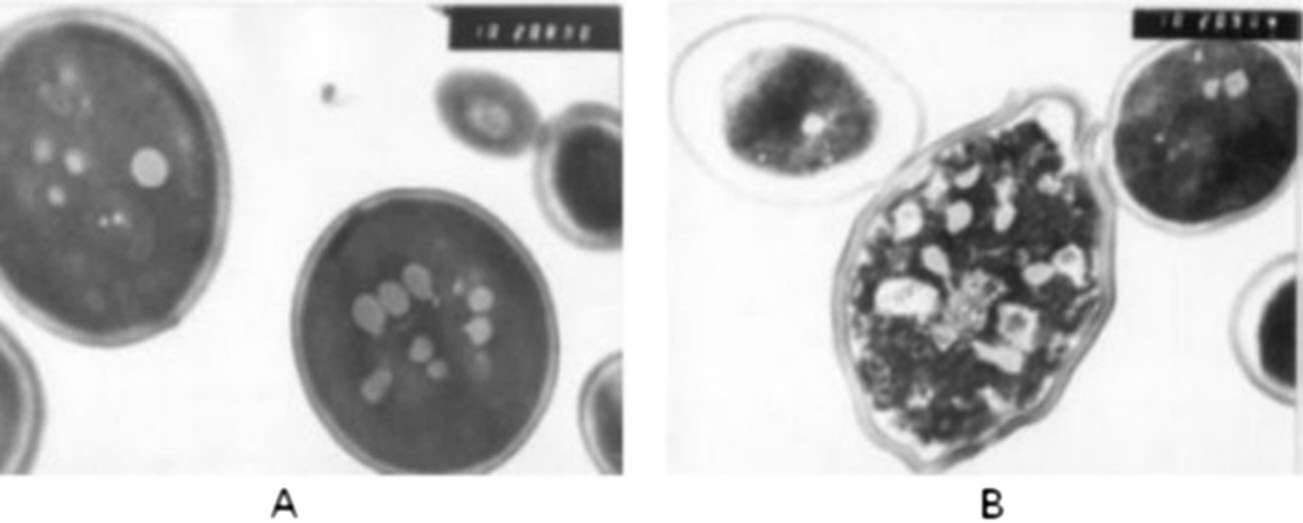
The electron microscope of yeast cell before ((**a**), *P* = 0.1 MPa) and after ((**b**), *P* = 400 MPa) pressurization

It is notable that for such path of approaching the denaturation curve one can minimize the influence of the irreversible coagulation, always occurring for the classical, “thermal” pasteurization. It is notable that “death curves” of microorganisms portrayed in the *P*-*T*, strongly resemble plane by the curves in Fig. 2 [20]. Despite this fact, as the key reason of the “killing action” of high pressures on microorganisms is recognized the action on more complex structure, mainly the cell wall. Figure 3 shows the interruption of cell walls in tested yeast cells. The notable impact of the high pressure on intracellular structures is also visible.

**Fig. 4:**
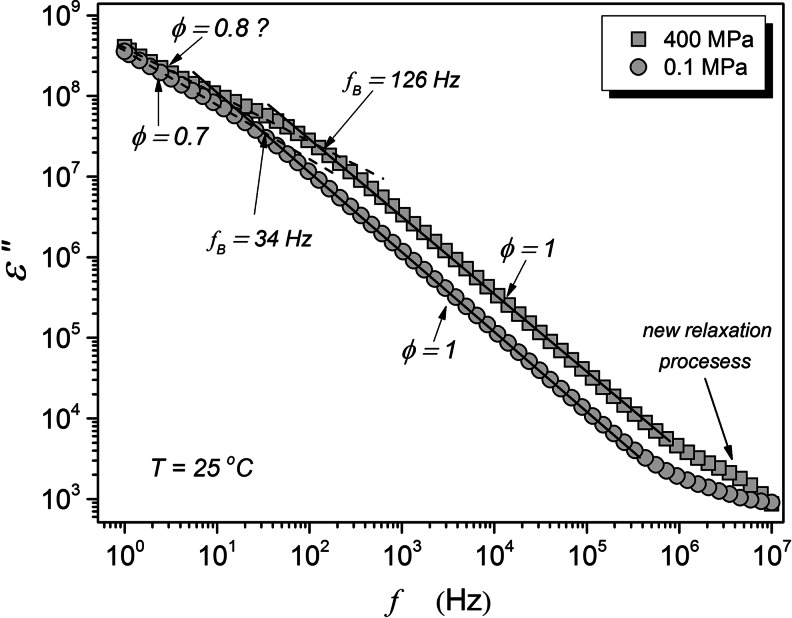
The imaginary part of dielectric permittivity in native (atmospheric pressure) and pressurized yeast for T = 25 °C isotherm. The key parts of plots follow the dependence *ε* ' ' (*f*) ∝ *f* ^− *ϕ*^, i.e. log_10_ *ε* ' ' (*f*) ∝ − *ϕ* log_10_ *f*. The frequency *f*_*B*_ denotes the crossover to the Maxwell – Wegner effect-dominated region

The question arises, how the pressure-induced destruction of cells manifests in the BDS monitoring. This report focuses on two basic characteristics present in the BDS output. The first one is the imaginary part of dielectric permittivity, shown in Fig. 4. On decreasing of the measurement frequency *ε* ' ' (*f*) values increases linearly. This process is described by *ε* ' ' (*f*) ∝ *f* ^− *ϕ*^ with the exponent *ϕ* = 1, as shown in Fig. 4. For *f* < 10*Hz* at *P* = 0.1*MPa* and *f* < 100*Hz* at *P* = 0.1*MPa*, the impact of the ionic polarizability emerges. On further decreasing of the frequency the evolution of log_10_ *ε* ' (*f*) vs. log_10_ *f* becomes progressively nonlinear, until reaching the domain described by the exponent *S* < 1 for lower frequencies. [6]. These changes can be related to the so strong impact of permanent “ionic impurities” that under the external electric field from the BDS spectrometer causes the parasitic polarization of plates of the capacitor. It is notable that this phenomenon appear for much higher frequencies for the pressurized yeast which indicates much higher content of “ionic impurities”, presumably associated with free ions of Na, K, Ca, Mg, Cl and molecular species related to organic acids [8].

It is also notable that for the pressurized yeast, a primary relaxation process with the peak at ca. 8 MHz emerges.

**Fig. 5:**
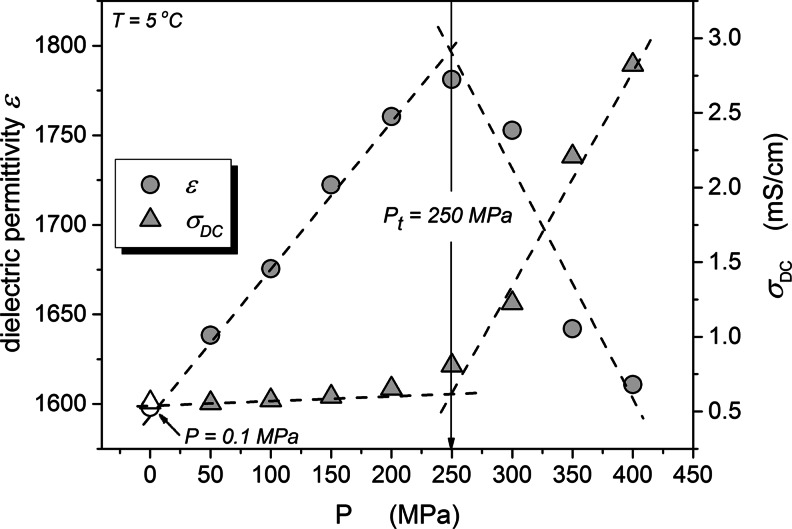
The pressure evolution of the real part of dielectric permittivity and the DC conductivity in yeast at T = 5 °C

**Fig. 6:**
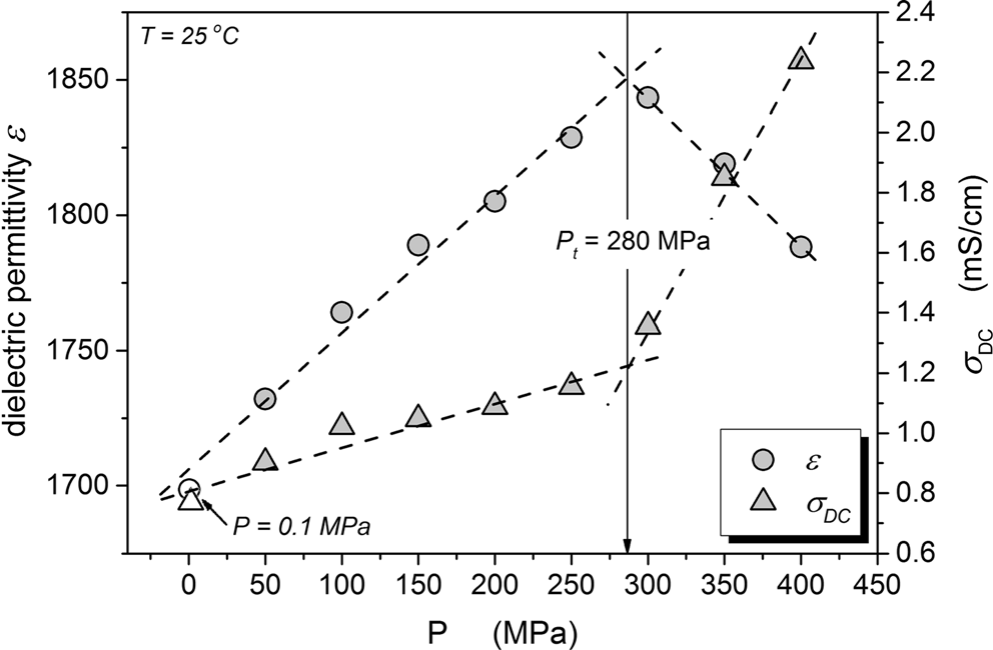
The pressure application time (*P* = 400 MPa) evolution of the real part of dielectric permittivity and the DC conductivity in yeast at T = 25 °C.

Figures 5 and 6 show the pressure dependence of the static dielectric permittivity and DC electric conductivity as the function of pressure at 5 and 25 °C temperatures. The evolution of the DC conductivity shows a clear evidence for a “threshold”, near *P*_*t*_ = 200 MPa for *T* = 5 °C and *P*_*t*_ = 300 MPa for *T* = 25 °C. Below *P*_*t*_ the increase of pressure increases the electric conductivity and above *P*_*t*_ further compression decreases the conductivity. For *P* < *P*_*t*_ the behavior is opposite to the one observed in “normal” liquids, where the pressurization decreases the electric conductivity, what is coupled with a notable increase of viscosity. The rise of conductivity *σ*(*P*) on increasing pressure up to *P*_*t*_ indicates the appearance of new free ions in the system, gradually released from a “reservoir of free ions”. This can be associated only with the break of cell walls and leakage of the intracellular fluid. Above the threshold pressure, for *P* > *P*_*t*_, the evolution of electric conductivity is similar to the behavior observed in “normal liquids” where number of free ions is constant and does not change during the experiment [6, 15, 17]. This indicates that sources of newly released free ions have been exhausted. This domain is described by the Barus equation as discussed above and shown in Fig. 5. These threshold pressures are also clearly visible in behavior of the dielectric constant *ε* ' (*P*) = *ε*(*P*). Up to *P* = *P*_*t*_ the linear increase of *ε* ' (*P*) is very weak and in fact close to *ε*(*P*) ≈ *const*, what suggests the virtual lack of the preferable arrangement of permanent dipole moment. For *P* > *P*_*t*_ dielectric constant *ε* ' (*P*) strongly increases indicating the creation of a notable parallel arrangement of permanent dipole moments. It is worth stressing that “threshold pressures” values manifest clearly both for *ε*(*P*) and *σ*(*P*) evolutions.

**Fig. 7:**
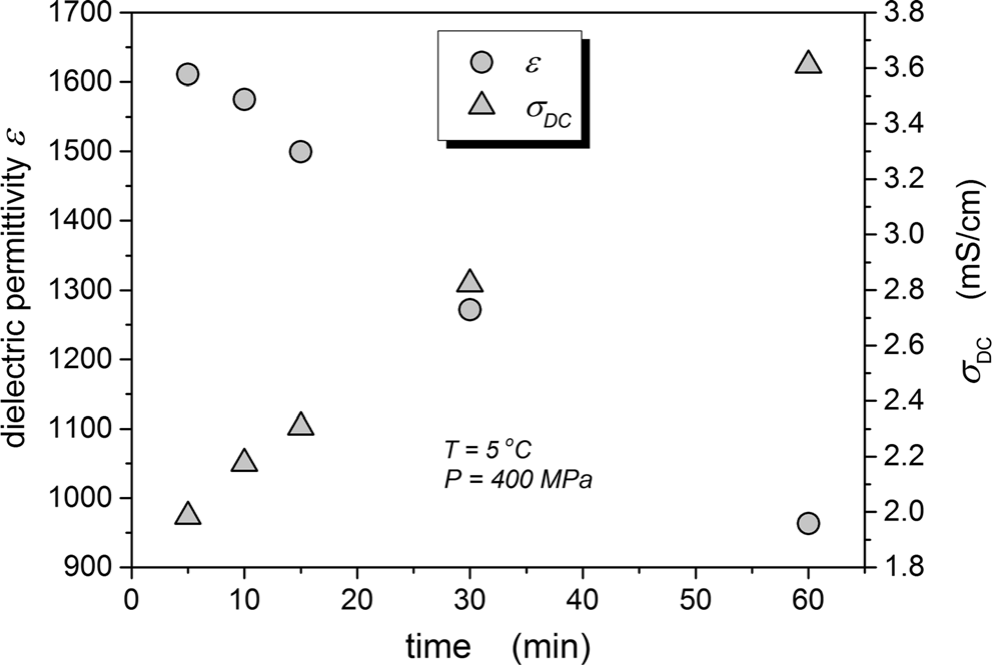
The time evolution of the DC electric conductivity and the static dielectric permittivity in the pressurized yeast at T = 5 °C

Figure 7 shows the impact of time on the pressurized yeast, monitored by the changes of *ε*(*P*) and *σ*(*P*). After 1 hour of high pressure acting, the electric conductivity increases and the dielectric constant decreases – by ca. 65 % of the initial value. These results indicate that despite different nature of processes monitored by dielectric constant and electric conductivity, they are strongly coupled.

**Fig. 8:**
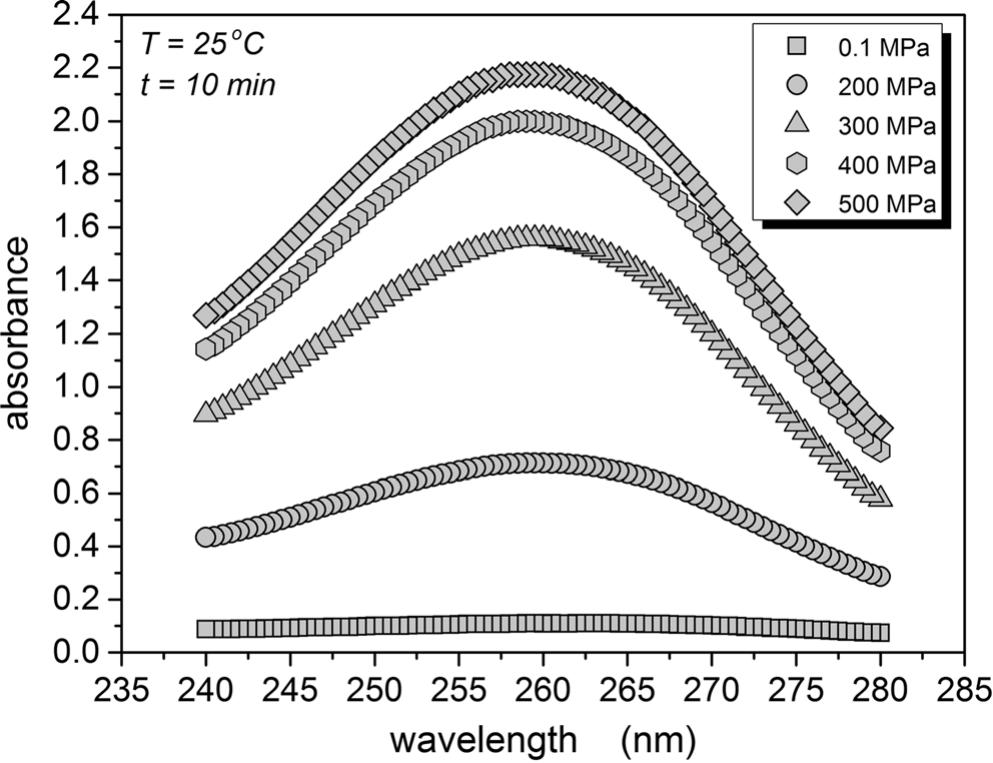
The UV absorbance of supernatant leaking out from cells of S. cerevisiae after pressurizing from 0.1 to 500 MPa

**Fig. 9:**
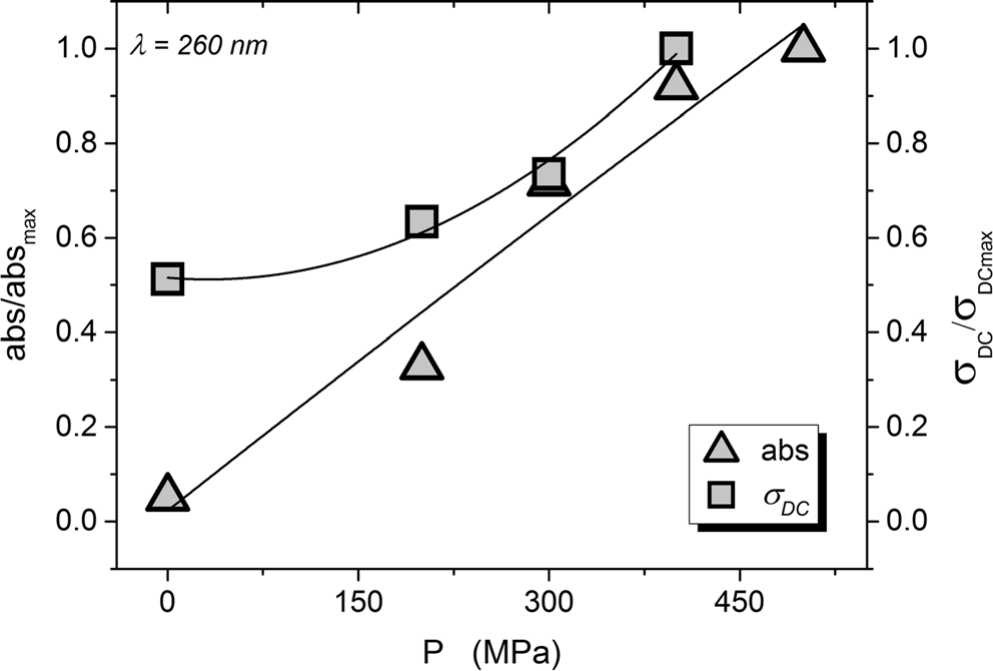
The correlation between absorbance at 260 nm of the supernatant after pressurizing of Saccharomyces cerevisiae and electric DC conductivity

The increase of the electric conductivity can be linked to the leakage of intracellular liquid, appearing due to the pressure-induced breaks of the cellular walls. One of methods to estimate the amount of the intracellular, naturally turbid, liquid, are spectrophotometric measurements of absorbance of clear supernatant at 260 nm after the removal of cells and cells’ debris by centrifugation. Figure 8 presents spectrophotometric results for the gradually compressed yeast. Figure 9 shows the clearly correlation between the normalized absorbance and the DC conductivity on increasing pressure in tested samples.

## Conclusions

Results presented show that the evolution of the static dielectric permittivity and particularly the DC electric conductivity can be considered as notable and sensitive tools for testing characteristics of pressurized yeast cells. It is notable that BDS experiments under pressure can yield high resolution results, hardly available by other physical method implemented for such challenging conditions. Microbiological studies of pressurized solutions of yeast and other model cells indicated that conditions related to the pasteurization, i.e. the reduction of the number of microorganisms by at least five decades (“*5 log*-*units*”), needs at least 400 MPa for minimum 3 minutes [8–11]. Moreover, “deeper” pasteurization takes place for pressurization under higher temperature. This paper lead to the conclusion that the break of cell walls may take place for lower pressure at lower temperatures (5 °C), already at 200–300 MPa. The effective process needs also more time than 3–5 minutes used in HPP technology. All these may suggest that the action of pressure leading to the “cold pasteurization” phenomenon for commercially most often used pressures *P* = 400–600 MPa is associated not only with the break of cell walls but also the influence of intracellular structures and pressure-induced denaturation.
